# Marital status as an independent prognostic factor in male breast cancer: a SEER-based cohort study

**DOI:** 10.1007/s12282-025-01733-3

**Published:** 2025-06-16

**Authors:** Ahmet Necati Sanli, Bilal Turan, Deniz Esin Tekcan Sanli, Fatih Aydogan, M Kadri Altundag

**Affiliations:** 1 Department of General Surgery, Abdulkadir Yuksel State Hospital, Gaziantep, Turkey; 2 Department of General Surgery, Faculty of Medicine, Suleyman Demirel University, Isparta, Turkey; 3 Department of Radiology, Faculty of Medicine, Gaziantep University, Gaziantep, Turkey; 4 Breast Health Center, Memorial Bahcelievler Hospital, Istanbul, Turkey; 5 MKA Breast Cancer Clinic, Ankara, Turkey

**Keywords:** Male breast cancer, Marital status, Survival, SEER, Prognostic factor, Psychosocial support

## Abstract

**Background:**

Male breast cancer (MBC) is a rare malignancy, and research on its prognostic factors remains limited. Marital status has been identified as a prognostic determinant in various cancers; however**,** the impact of marital status on MBC survival outcomes remains understudied**.** This study examines the relationship between marital status and survival in MBC patients using a large, population-based cohort.

**Methods:**

A retrospective cohort study was conducted using the Surveillance, Epidemiology, and End Results (SEER) 17 Registries database (2010–2021). MBC patients (*n* = 5,994) were categorized into four marital status groups: married, unmarried, divorced, and widowed**.** Kaplan–Meier survival curves, log-rank tests, and Cox proportional hazards models were used to assess overall survival (OS) and disease-specific survival (DSS).

**Results:**

Significant differences in OS and DSS were observed among marital status groups (*p* < 0.001). Widowed patients had the poorest survival outcomes, with a 2.59 times higher increased mortality risk (HR = 2.594, 95% CI: 2.246–2.996, *p* < 0.001) compared to married individuals in univariate analysis. This association remained significant in multivariate analysis, with widowed patients demonstrating a 1.72 times higher mortality risk (HR = 1.724, 95% CI: 1.421–2.092, *p* < 0.001) after adjustment for clinicopathological and demographic variables. Unmarried and divorced patients also had poorer survival outcomes than married patients, albeit with lower hazard ratios.

**Conclusions:**

Marital status is an independent prognostic factor in MBC, with widowed patients experiencing the poorest outcomes. These findings underscore the important role of social and psychological support in cancer prognosis. Integrating psychosocial support programs and patient-centered care approaches into oncological practice can help reduce survival disparities**.**

## Introduction

Male breast cancer (MBC) is a rare malignancy, accounting for approximately 1% of all breast cancer cases [1]. Due to its rarity, research on prognostic factors affecting MBC outcomes remains limited. Studies examining the impact of psychosocial factors on cancer survival in this patient group are particularly scarce [1].

The biological factors that determine survival in the majority of cancer types are now better understood; however, the impact of psychosocial factors, such as marital status, on survival in cancer patients remains largely unclear [2]. Similarly, in breast cancer, clinicopathological features such as subtypes, AJCC TNM stages, tumor size, and treatment strategies have been investigated as prognostic factors [3, 4]. However, social factors are increasingly emphasized in the progression of these diseases [5].

Marital status has been shown to play an independently significant role in the prognosis of various carcinomas, such as pancreatic carcinoma, prostate carcinoma, lung carcinoma, and colorectal carcinoma, with married patients demonstrating better survival rates. [5–8]. Despite breast cancer being the most common malignancy among women, studies focusing on its association with marital status remain limited. Single breast cancer patients have a higher total mortality rate compared to married patients; the relationship varies by race/ethnicity, tumor subtype, and social status. [9]. Additionally, the understanding and investigation of the impact of marital status on MBC survival rates remain limited. Although marital status is recognized as an important factor for survival in several cancers, its effect on MBC has not been adequately studied. [5–9].

Marital status has been shown to play an independently significant role in the prognosis of various carcinomas, such as pancreatic carcinoma, prostate carcinoma, lung carcinoma, and colorectal carcinoma, with married patients demonstrating better survival rates [5–8]. Despite breast cancer being the most common malignancy among women, studies focusing on its association with marital status remain limited. Single breast cancer patients have a higher total mortality rate compared to married patients; the relationship varies by race/ethnicity, tumor subtype, and social status [9]. Additionally, understanding and exploration of the impact of marital status on MBC survival rates have remained limited. Although marital status is recognized as an important factor for survival in several cancers, its effect on MBC has not been adequately studied [5–9].

Epidemiological studies show that married cancer patients generally have better outcomes compared to unmarried, divorced, or widowed patients [5, 10]. Possible explanations include increased social support, better treatment adherence, and improved access to healthcare among married individuals [5, 10]. Being married is significantly associated with better overall survival. Unmarried men are linked to poorer survival outcomes. However, whether this association extends to MBC remains unclear. Marital status might be considered when providing cancer care [5, 10].

Marital status has been identified as an independent prognostic factor associated with better survival rates in various cancer types, likely due to biological, psychological, or socioeconomic factors [9]. The impact of social support provided by a partner may be physiologically mediated through neuroendocrine, neural, and immune interactions directly related to cancer. For instance, cancer patients with higher-quality social support exhibit increased activity of natural killer (NK) cells, key cytotoxic immune cells that can recognize and destroy cancer cells. Additionally, the hormone oxytocin, released during social interactions, may indirectly inhibit cancer cell growth by suppressing the stress response [9, 10].

This study aims to evaluate the relationship between marital status and survival in male breast cancer (MBC) patients using a large, population-based dataset (SEER). By identifying disparities in survival outcomes, it is believed to provide insights into potential interventions that could mitigate the impact of marital status on cancer prognosis. This study will offer critical data to better understand the influence of marital status on MBC survival and contribute to clinical decision-making processes.

This study aims to evaluate the relationship between marital status and survival in MBC patients using a large, population-based dataset (SEER). By identifying disparities in survival outcomes, it is expected to provide insights into potential interventions that could mitigate the impact of marital status on cancer prognosis. This study will offer valuable data to better understand the influence of marital status on MBC survival and contribute to clinical decision-making processes.

## Materials and methods

### Study population

This retrospective cohort study utilized data from the SEER 17 Registries database, covering cases from 2010 to 2021. The study included male breast cancer patients (*N* = 5994). Patients with unknown marital status (*n* = 372) and those with missing data on key variables were excluded from the analysis.

Marital status was categorized into four groups: married, unmarried, divorced, and widowed. The Unmarried group included individuals who were single (never married) or cohabiting (in a domestic partnership), while the Divorced group included both divorced and separated individuals.

Patients were categorized into three age groups: those younger than 50 years, between 50 and 64 years, and 65 years or older. Race was classified into White, Black, and Other, with American Indian/Alaska Native and Asian/Pacific Islander grouped under the"Other"category. Median household income was categorized as < $50,000, $50,000–$75,000, and > $75,000 based on SEER census-linked county-level data, in accordance with thresholds commonly used in population-based studies. Tumor location was recorded based on its anatomical site in the breast, including the lower inner quadrant, lower outer quadrant, upper inner quadrant, upper outer quadrant, and overlapping lesions. Laterality of the tumor (left or right breast) was also documented. Additionally, patients'residential areas were classified using the Rural–Urban Continuum Code, which distinguishes metropolitan from non-metropolitan counties to assess the impact of urbanization on health outcomes.

Patients were further categorized by histological classification, which included invasive ductal carcinoma and other cancer types. Estrogen receptor (ER) and progesterone receptor (PR) statuses were reported as positive or negative, while HER2 status was classified similarly. Molecular subtypes were categorized as hormone receptor-positive/HER2-negative, hormone receptor-positive/HER2-positive, hormone receptor-negative/HER2-positive, and hormone receptor-negative/HER2-negative. Tumor grade, tumor size (T classification), and nodal involvement (N classification) were also recorded. AJCC stage was recorded as reported in the SEER database. The staging system edition varied by year of diagnosis: the 6th edition was used for cases diagnosed between 2010 and 2015, the 7th edition for cases diagnosed from 2016 to 2017, and the 8th edition for those diagnosed from 2018 onwards, in accordance with SEER registry protocols. Surgical treatments were categorized into breast-conserving surgery, mastectomy, or no surgery. Radiotherapy and chemotherapy administrations were recorded as performed or not performed. Systemic therapy was defined as any systemic treatment administered, including chemotherapy, hormonal therapy, targeted therapy, or a combination thereof. In the SEER database, this variable is recorded as a binary indicator ("yes"or"no"), without further subclassification regarding the specific type or regimen of systemic treatment. Therefore, our categorization reflects the available binary coding within SEER. Metastatic status at diagnosis was recorded for the brain, bone, liver, and lung, indicating the presence or absence of metastasis in these organs. This study utilized de-identified data from the SEER database, which is publicly available and maintains patient anonymity; therefore, ethical approval and informed consent were not required.

### Statistical analysis

All statistical analyses were conducted using R (version 4.2.2) and IBM SPSS Statistics (version 29) software. Descriptive statistics were used to summarize demographic and clinical characteristics. Categorical variables were presented as frequencies and percentages, while continuous variables were expressed as means and standard deviations. To compare categorical variables between groups, the Pearson Chi-Square test was employed. Since the age variable did not follow a normal distribution, the Kruskal–Wallis test was used to compare the age distributions across the four marital status groups. Post hoc pairwise comparisons were conducted using the Games-Howell test to assess significant differences between individual groups. The Games-Howell test was chosen as the post hoc method because it does not assume equal variances and is appropriate for comparing groups with unequal sample sizes, which was observed among the marital status groups in this study.

Survival analyses were conducted using the Kaplan–Meier method to estimate overall survival (OS) and disease-specific survival (DSS). The log-rank test was applied to compare survival distributions between groups. Both univariate and multivariate analyses were performed using Cox proportional hazards regression models to identify factors associated with survival outcomes, with results reported as hazard ratios (HRs) and 95% confidence intervals (CIs). A *p*-value of < 0.05 was considered statistically significant.

## Results

### Baseline characteristics

This study included 5,994 male breast cancer (MBC) patients, categorized based on their marital status as Married, Unmarried, Divorced, and Widowed (Table 1). The mean age of the study population was 67.49 ± 12.59 years, with significant differences among marital status groups (*p* < 0.001, Kruskal–Wallis test). Post hoc analysis showed that all pairwise comparisons were statistically significant (*p* < 0.001). Widowed patients had the highest mean age (78.74 ± 9.62 years), while unmarried patients had the lowest (60.57 ± 13.16 years). Age distribution also varied significantly, with the unmarried group having the highest proportion of patients younger than 50 years (19.8%) and the widowed group having the lowest (0.5%). Most widowed (90.8%) and married (64.0%) patients were aged ≥ 65 years, whereas the unmarried group had the lowest proportion (39.6%). Among married individuals, the mean age was 68.21 years, with 7.17% under 50 years of age and 92.83% aged 50 or older (Table 1).

**Table 1:** Clinicopathological characteristics of all patients

		Married	Unmarried	Divorced	Widowed	
		n (%)	n (%)	n (%)	n (%)	*P*
Age (Mean ± SD)		68.21 ± 11.86	60.57 ± 13.16	65.66 ± 10.86	78.74 ± 9.62	** < *0.001***
	≤ 50	271 (7.2)	186 (19.6)	37 (7.5)	2 (0.5)	** < *0.001***
	50–64	1089 (28.8)	381 (40.6)	173 (35.2)	36 (8.7)	
	65 ≥	2419 (64)	371 (39.6)	281 (57.2)	376 (90.8)	
Race	White	3102 (82.1)	630 (67.2)	378 (77)	342 (82.6)	** < *0.001***
	Black	411 (10.9)	247 (26.3)	93 (18.9)	54 (13.1)	
	Other	241 (6.4)	54 (5.8)	16 (3.3)	18 (4.3)	
	Unknown	25 (0.7)	7 (0.7)	4 (0.8)	0	
Place of Residence	Metro ≥ 1 M	2342 (62)	589 (62.8)	274 (55.8)	222 (53.6)	** < *0.001***
	Metro 250 K-1 M	779 (20.6)	199 (21.2)	108 (22)	101 (24.4)	
	Metro < 250 K	265 (7)	56 (6)	39 (7.9)	38 (9.2)	
	Non-Metro Adj	250 (6.6)	67 (7.1)	38 (7.7)	21 (5.1)	
	Non-Metro Non-Adj	143 (3.8)	27 (2.9)	32 (6.5)	32 (7.7)	
Median Houesehold Income	< $50,000	386 (10.2)	120 (12.8)	77 (15.7)	43 (10.4)	** < *0.001***
	$50,000-$75,000	1135 (30)	286 (30.5)	170 (34.6)	158 (38.2)	
	> $75,000	2258 (59.8)	532 (56.7)	244 (49.7)	213 (51.4)	
Location	Central	1788 (47.3)	414 (44.1)	231 (47)	201 (48.6)	** < *0.001***
	Upper-outer quadrant	470 (12.4)	92 (9.8)	67 (13.6)	46 (11.1)	
	Upper-inner quadrant	153 (4)	40 (4.3)	13 (2.6)	14 (3.4)	
	Lower-outer quadrant	127 (3.4)	30 (3.2)	13 (2.6)	11 (2.7)	
	Lower-inner quadrant	71 (1.9)	13 (1.4)	6 (1.2)	11 (2.7)	
	Overlapping	593 (15.7)	158 (16.8)	71 (14.5)	52 (12.6)	
	Unknown	577 (15.3)	191 (20.4)	90 (18.3)	79 (19.1)	
Laterality	Right	1757 (46.5)	424 (45.2)	225 (45.8)	183 (44.2)	** < *0.001***
	Left	1976 (52.3)	487 (51.9)	250 (50.9)	215 (51.9)	
	Bilateral	52.3%	51.9%	50.9%	51.9%	
	Unknown	46 (1.2)	27 (2.9)	16 (3.3)	16 (3.9)	
Sequence	Only one primary cancer	2470 (65.4)	700 (74.6)	324 (66)	236 (57)	** < *0.001***
	2 or more primary cancer	1309 (34.6)	238 (25.4)	167 (34)	178 nn	
Histology	IDC	3320 (87.9)	785 (83.7)	398 (81.1)	342 (82.6)	
	Others	459 (12.1)	153 (16.3)	93 (18.9)	72 (17.4)	
Grad	1	408 (10.8%)	89 (9.5%)	50 (10.2%)	45 (10.9%)	< 0.001
	2	1834 (48.5%)	435 (46.4%)	216 (44.0%)	162 (39.1%)	
	3	1191 (31.5%)	280 (29.9%)	163 (33.2%)	135 (32.6%)	
	Unknown	346 (9.2%)	134 (14.3%)	62 (12.6%)	72 (17.4%)	
ER	Positive	3509 (92.9%)	846 (90.2%)	443 (90.2%)	363 (87.7%)	< 0.001
	Negative	105 (2.8%)	28 (3.0%)	18 (3.7%)	14 (3.4%)	
	Unknown	165 (4.4%)	64 (6.8%)	30 (6.1%)	37 (8.9%)	
PR	Positive	3255 (86.1%)	774 (82.5%)	417 (84.9%)	336 (81.2%)	< 0.001
	Negative	346 (9.2%)	91 (9.7%)	43 (8.8%)	34 (8.2%)	
	Unknown	178 (4.7%)	73 (7.8%)	31 (6.3%)	44 (10.6%)	
HER2	Negative	401 (10.6%)	126 (13.4%)	54 (11.0%)	40 (9.7%)	< 0.001
	Positive	3078 (81.5%)	705 (75.2%)	378 (77.0%)	309 (74.6%)	
	Unknown	300 (7.9%)	107 (11.4%)	59 (12.0%)	65 (15.7%)	
Molecular subtype	Luminal A	2999 (79.4%)	689 (73.5%)	364 (74.1%)	301 (72.7%)	< 0.001
	Luminal B	381 (10.1%)	115 (12.3%)	52 (10.6%)	37 (8.9%)	
	HER2 rich	20 (0.5%)	11 (1.2%)	2 (0.4%)	3 (0.7%)	
	TNBC	72 (1.9%)	13 (1.4%)	14 (2.9%)	8 (1.9%)	
	Unknown	307 (8.1%)	110 (11.7%)	59 (12.0%)	65 (15.7%)	
T stage	T1	1644 (43.5%)	308 (32.8%)	179 (36.5%)	144 (34.8%)	< 0.001
	T2	1537 (40.7%)	370 (39.4%)	195 (39.7%)	163 (39.4%)	
	T3	99 (2.6%)	56 (6.0%)	18 (3.7%)	19 (4.6%)	
	T4	276 (7.3%)	116 (12.4%)	48 (9.8%)	46 (11.1%)	
	Unknown	223 (5.9%)	88 (9.4%)	51 (10.4%)	42 (10.1%)	
N stage	N0	2098 (55.5%)	461 (49.1%)	236 (48.1%)	223 (53.9%)	< 0.001
	N1	1142 (30.2%)	297 (31.7%)	163 (33.2%)	120 (29.0%)	
	N2	250 (6.6%)	70 (7.5%)	39 (7.9%)	23 (5.6%)	
	N3	150 (4.0%)	52 (5.5%)	24 (4.9%)	12 (2.9%)	
	Unknown	139 (3.7%)	58 (6.2%)	29 (5.9%)	36 (8.7%)	
AJCC Stage	1	1695 (44.9%)	333 (35.5%)	183 (37.3%)	163 (39.4%)	< 0.001
	2	1170 (31.0%)	270 (28.8%)	143 (29.1%)	120 (29.0%)	
	3	477 (12.6%)	144 (15.4%)	68 (13.8%)	55 (13.3%)	
	4	266 (7.0%)	137 (14.6%)	60 (12.2%)	41 (9.9%)	
	Unknown	171 (4.5%)	54 (5.8%)	37 (7.5%)	35 (8.5%)	
Bone metastasis	Yes	174 (4.6%)	102 (10.9%)	36 (7.3%)	24 (5.8%)	< 0.001
	No	3530 (93.4%)	812 (86.6%)	444 (90.4%)	367 (88.6%)	
	Unknown	75 (2.0%)	24 (2.6%)	11 (2.2%)	23 (5.6%)	
Brain metastasis	Yes	20 (0.5%)	8 (0.9%)	8 (1.6%)	2 (0.5%)	< 0.001
	No	3681 (97.4%)	905 (96.5%)	471 (95.9%)	389 (94.0%)	
	Unknown	78 (2.1%)	25 (2.7%)	12 (2.4%)	23 (5.6%)	
Liver metastasis	Yes	36 (1.0%)	19 (2.0%)	6 (1.2%)	7 (1.7%)	< 0.001
	No	3667 (97.0%)	896 (95.5%)	473 (96.3%)	383 (92.5%)	
	Unknown	76 (2.0%)	23 (2.5%)	12 (2.4%)	24 (5.8%)	
Lung metastasis	Yes	108 (2.9%)	551″” (5.9%)	22 (4.5%)	21 (5.1%)	< 0.001
	No	3593 (95.1%)	859 (91.6%)	458 (93.3%)	369 (89.1%)	
	Unknown	78 (2.1%)	24 (2.6%)	11 (2.2%)	24 (5.8%)	
Surgery	BCS	75 (2.0%)	18 (1.9%)	9 (1.8%)	6 (1.4%)	< 0.001
	Mastectomy	2878 (76.2%)	625 (66.6%)	360 (73.3%)	241 (58.2%)	
	Not performed	406 (10.7%)	197 (21.0%)	74 (15.1%)	106 (25.6%)	
	Unknown	420 (11.1%)	98 (10.4%)	48 (9.8%)	61 (14.7%)	
Radiotherapy	Yes	1118 (29.6%)	256 (27.3%)	159 (32.4%)	93 (22.5%)	< 0.001
	No	2661 (70.4%)	682 (72.7%)	332 (67.6%)	321 (77.5%)	
Chemotherapy	Yes	1335 (35.3%)	389 (41.5%)	188 (38.3%)	78 (18.8%)	< 0.001
	No	2444 (64.7%)	549 (58.5%)	303 (61.7%)	336 (81.2%)	
Systemic therapy	Yes	2625 (69.5%)	584 (62.3%)	315 (64.2%)	193 (46.6%)	< 0.001
	No	1154 (30.5%)	354 (37.7%)	176 (35.8%)	221 (53.4%)	

Significant racial differences were observed among marital status groups (*p* < 0.001). White patients were more prevalent among the married (82.1%) and widowed (82.6%) groups, while the unmarried group had the lowest proportion of White patients (67.2%) and the highest proportion of Black patients (26.3%). The proportion of Black patients was also higher in the divorced group (18.9%) compared to married patients. Residence and income levels varied across groups, with widowed patients more likely to reside in non-metropolitan counties (7.7%) and having the highest proportion of low-income individuals (38.2%), while married patients were more likely to reside in urban areas and had the highest percentage in the highest income bracket (59.8%) (Table 1).

### Tumor characteristics

Tumor localization differed significantly among marital status groups (*p* = 0.015), with central localization being the most common site in all groups. Unmarried patients had a significantly higher proportion of tumors classified as"Not Otherwise Specified (NOS)"(20.4%) compared to married patients (15.3%). Laterality showed variation, with 45.86% of cases involving the right side, 51.58% the left side, 1.36% bilateral involvement, and 1.20% unspecified laterality.

Histological subtype analysis revealed that invasive ductal carcinoma was the most common type in all groups but was significantly more frequent in married patients (87.9%) compared to unmarried (83.7%) and divorced (81.1%) patients. Tumor grade also varied significantly among marital status groups (*p* < 0.001). Low-grade (Grade 1–2) tumors were more common in married patients (56.2%), whereas high-grade (Grade 3–4) tumors were more frequent in unmarried patients (51.6%).

Hormone receptor status showed significant variations (p < 0.001). Estrogen receptor (ER)-positive tumors were most common among married patients (92.9%) and least frequent among widowed patients (87.7%). A similar pattern was observed for progesterone receptor (PR)-positive tumors, with the highest frequency in married patients (86.1%) and the lowest in widowed patients (81.2%). HER2-negative tumors were predominant across all groups but were most common in married patients (81.5%) and less frequent in unmarried patients (75.2%). Conversely, HER2-positive tumors were slightly more common in unmarried patients (13.4%) compared to married patients (10.6%).

### Cancer stage and treatment modalities

Significant differences were found in cancer stage at diagnosis (*p* < 0.001). Early-stage disease (Stage I–II) was more frequently observed in married patients (68.7%), whereas unmarried patients had a higher proportion of advanced-stage disease (Stage III–IV, 30.9%). Tumor size analysis indicated that smaller tumors (T1) were more common in married patients (48.5%), while larger tumors (T3–T4) were more prevalent among unmarried patients (21.3%). Lymph node involvement also varied, with married patients more likely to present with N0 status (62.4%) and unmarried patients more frequently exhibiting advanced nodal disease (N2–N3, 18.6%).

Treatment approaches also differed based on marital status. Mastectomy was the most common surgical procedure across all groups, with the highest rate observed among married patients (59.4%), while breast-conserving surgery was more frequently utilized in unmarried patients (29.8%). Widowed patients had the highest proportion of cases in which no surgery was performed (16.3%). Radiotherapy was most frequently administered to unmarried patients (44.3%) and least frequently to widowed patients (34.8%), who also had the highest proportion of patients not receiving radiotherapy (65.2%). Similarly, chemotherapy was most common among unmarried patients (43.2%) and least frequent in widowed patients (29.6%).

### Metastasis at diagnosis

Metastatic status at diagnosis significantly differed among marital status groups (*p* < 0.001). Unmarried patients had the highest proportion of metastases across all four sites (brain, bone, liver, lung) compared to other groups. Bone metastases were the most common, with the highest frequency observed in unmarried patients (55.4%), while brain metastases were the least common but were still more frequently reported among unmarried patients than in other groups (4.3%)**.** Conversely, widowed patients had the lowest rates of metastases at all sites. These metastasis rates were calculated among patients diagnosed with metastatic disease rather than the entire cohort, ensuring that the percentages reflect the distribution within the metastatic patient subgroup**.**

### Survival analyses

**Fig. 1:**
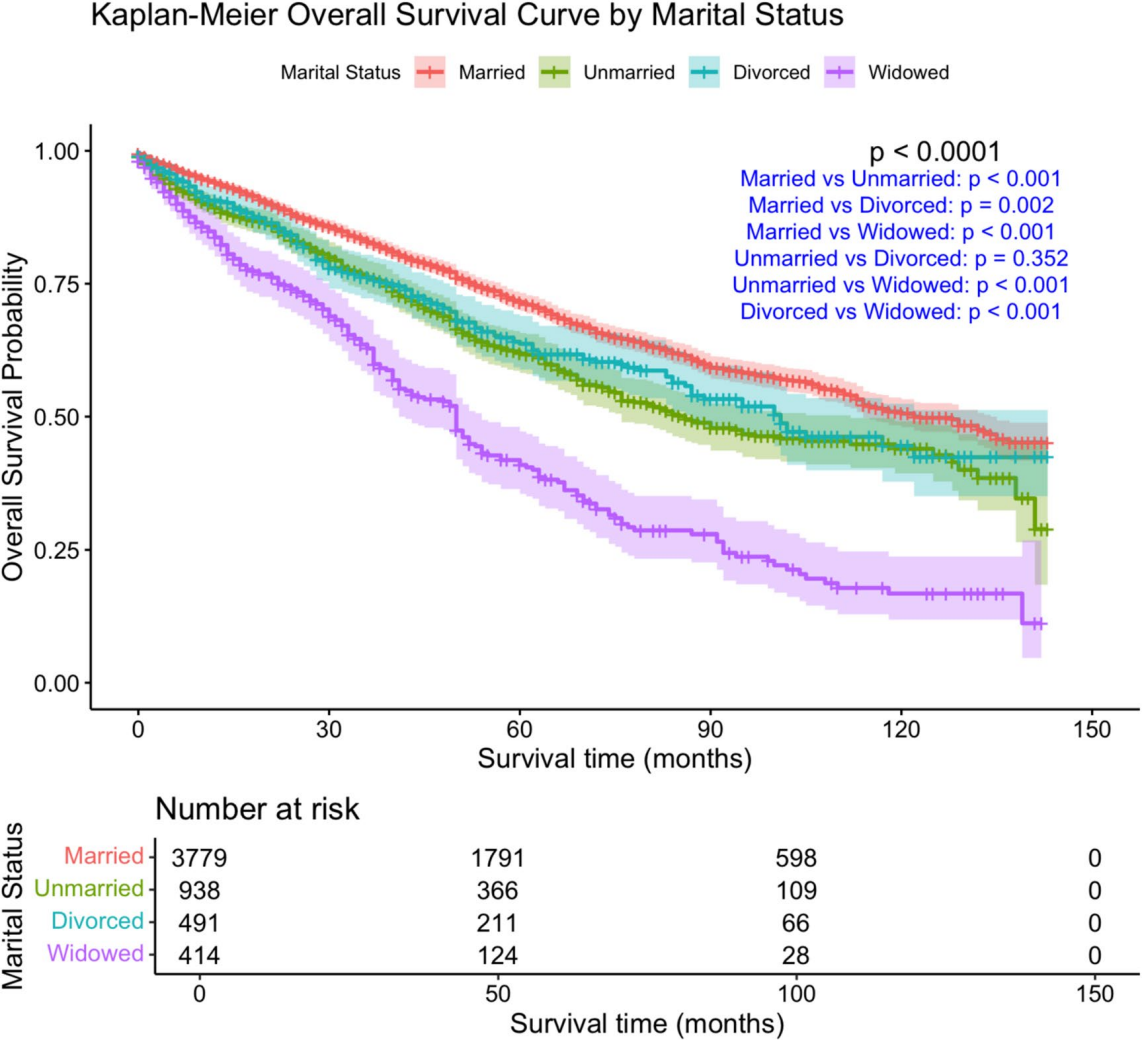
KM overall survival curve by marital status in MBC PATİENTS

Kaplan–Meier survival analysis demonstrated significant differences in overall survival (OS) among marital status groups (Fig. 1**)**. Widowed patients exhibited the worst survival outcomes. The results of univariate and multivariate Cox regression analyses for OS are presented in Table 2.

**Table 2:** Cox regression analysis according to overall survival

		Univariate analysis		Multivariate analysis	
		HR (95% CI)	*p*	HR (95% CI)	*p*
Marital status	Married	Reference	< 0.001	Reference	< 0.001
	Unmarried	1.40 (1.24–1.59)	< 0.001	1.41 (1.19–1.67)	< 0.001
	Divorced	1.29 (1.10–1.52)	0.002	1.31 (1.07–1.59)	0.009
	Widowed	2.59 (2.25–3.00)	< 0.001	1.72 (1.42–2.09)	< 0.001
Age	≤ 50	Reference	< 0.001	Reference	< 0.001
	50–64	1.22 (0.97–1.53)	0.079	1.28 (0.97–1.70)	0.085
	65 ≥	2.43 (1.97–2.99)	< 0.001	2.34 (1.78–3.08)	< 0.001
Race	White	Reference	< 0.001	Reference	0.120
	Black	1.19 (1.05–1.35)	0.007	1.03 (0.87–1.22)	0.765
	Other	0.71 (0.56–0.90)	0.004	.73 (0.54–0.99)	0.044
Place of Residence	Metro ≥ 1 M	Reference	0.006	Reference	0.110
	Metro 250 K-1 M	1.13 (1.01–1.27)	0.033	1.21 (1.05–1.40)	0.008
	Metro < 250 K	1.15 (0.96–1.37)	0.131	1.14 (0.90–1.46	0.272
	Non-Metro Adj	1.21 (1.01–1.45)	0.038	1.11 (0.86–1.44)	0.419
	Non-Metro Non-Adj	1.36 (1.11–1.68)	0.003	1.19 (0.87–1.62)	0.274
Median Houesehold Income	< $50,000	Reference	0.012	Reference	0.333
	$50,000-$75,000	1.23 (1.07–1.41)	0.004	1.13 (0.90–1.42)	0.297
	> $75,000	1.08 (0.98–1.20)	0.125	1.10 (0.96–1.25)	0.163
Sequence	Only one primary cancer	Reference		Reference	
	2 or more primary cancer	1.33 (1.21–1.46)	< 0.001	1.35 (1.19–1.52)	< 0.001
Histology	IDC	Reference		Reference	
	Others	1.47 (1.31–1.66)	< 0.001	.81 (0.64–1.04)	0.101
Grade	1	Reference	< 0.001	Reference	< 0.001
	2	1.15 (0.97–1.37)	0.120	1.05 (0.85–1.29)	0.650
	3	1.65 (1.39–1.97)	< 0.001	1.50 (1.21–1.86)	< 0.001
ER	Positive	Reference		Reference	
	Negative	1.80 (1.41–2.29)	< 0.001	1.75 (0.54–5.66)	0.350
PR	Positive	Reference		Reference	
	Negative	1.52 (1.32–1.77)	< 0.001	1.28 (1.04–1.59)	0.022
HER2	Negative	Reference		-	
	Positive	1.25 (1.08–1.44)	0.003	-	
Molecular subtype	Luminal A	Reference	< 0.001	Reference	0.051
	Luminal B	1.82 (1.63–2.04)	< 0.001	1.27 (1.07–1.52)	0.007
	HER2 rich	2.99 (2.38–3.76)	0.957	.74 (0.17–3.24)	0.687
	TNBC	3.43 (2.95–4.00)	< 0.001	.87 (0.24–3.09)	0.823
T stage	T1	Reference	< 0.001	Reference	< 0.001
	T2	1.82 (1.63–2.04)	< 0.001	1.68 (1.39–2.02)	< 0.001
	T3	2.99 (2.38–3.76)	< 0.001	2.12 (1.53–2.94)	< 0.001
	T4	3.43 (2.95–4.00)	< 0.001	1.66 (1.27–2.17	< 0.001
N stage	N0	Reference	< 0.001	Reference	0.681
	N1	1.28 (1.15–1.42)	< 0.001	1.08 (0.94–1.26)	0.283
	N2	1.40 (1.18–1.66)	< 0.001	1.12 (0.86–1.45)	0.408
	N3	1.69 (1.38–2.07)	< 0.001	1.14 (0.85–1.55)	0.386
AJCC Stage	1	Reference	< 0.001	Reference	< 0.001
	2	1.63 (1.44–1.84)	< 0.001	1.06 (0.86–1.31)	0.569
	3	2.11 (1.82–2.44)	< 0.001	1.57 (1.15–2.13)	0.004
	4	7.18 (6.21–8.31)	< 0.001	2.56 (1.87–3.49)	< 0.001
Surgery	BCS	Reference	< 0.001	Reference	< 0.001
	Mastectomy	1.06 (0.72–1.55)	0.778	1.17 (0.62–2.22)	0.636
	Not performed	5.30 (3.59–781)	< 0.001	3.06 (1.57–5.98)	0.001
Radiotherapy	Yes	Reference		Reference	
	No	1.31 (1.18–1.46)	< 0.001	1.14 (0.98–1.33)	0.093
Chemotherapy	Yes	Reference		Reference	
	No	1.46 (1.32–1.61)	< 0.001	1.52 (1.31–1.76)	< 0.001
Systemic therapy	Yes	Reference		Reference	
	No	2.28 (2.08–2.50)	< 0.001	1.19 (1.03–1.38)	0.016

**Fig. 2:**
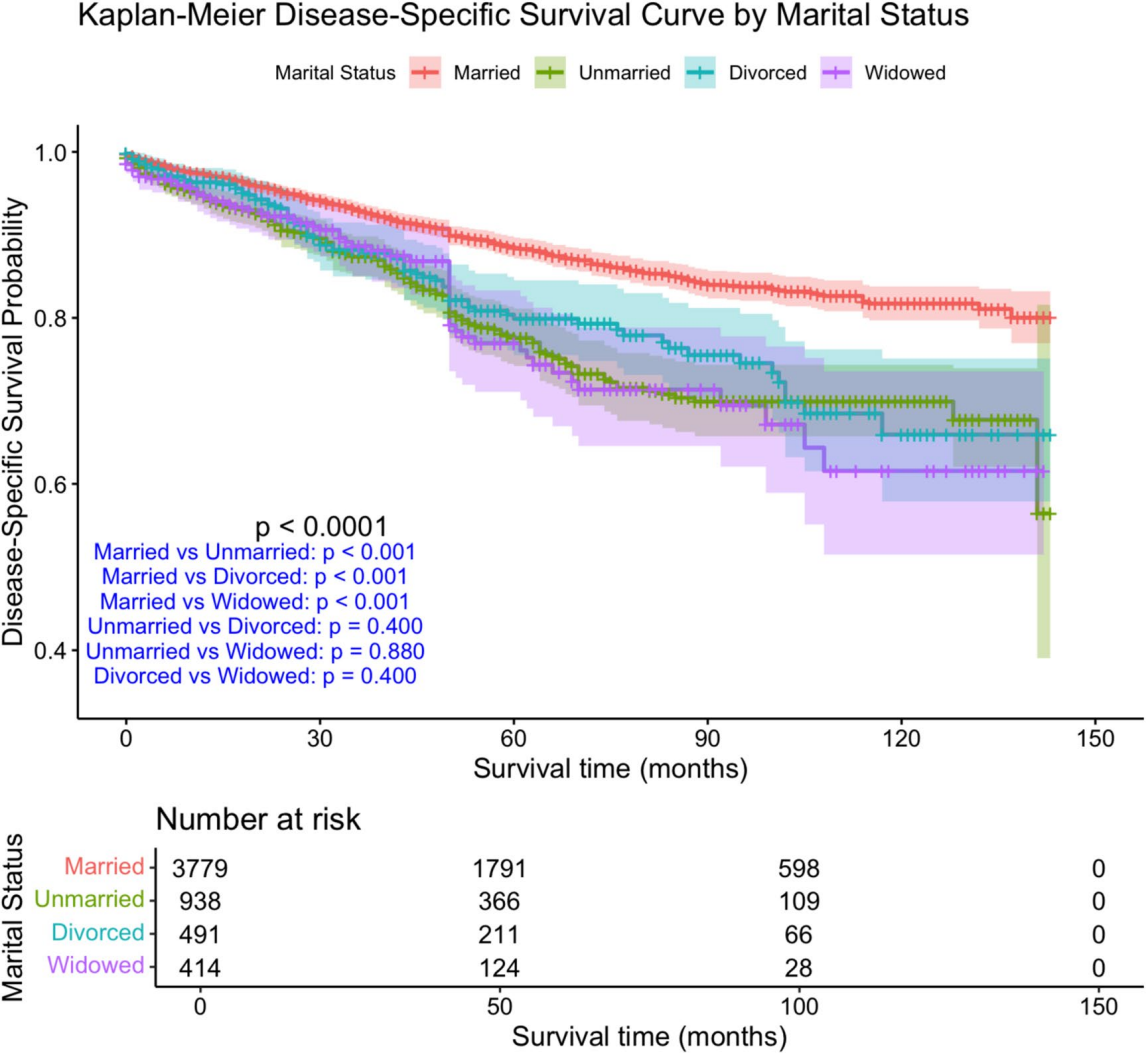
KM disease-specific survival curve by marital status in MBC PATİENTS

Significant differences in OS and DSS were observed among marital status groups (p < 0.001, log-rank test). (Figs. 1, 2). Married patients had the highest 5-year OS (71.5%), followed by divorced (63.7%) and unmarried (61.9%), whereas widowed patients had the lowest OS (40.9%). (Table 2). At 10 years, this survival gap widened, with OS rates of 50.6% in married, 44.5% in divorced, 43.9% in unmarried, and 16.8% in widowed patients. A similar trend was noted for DSS, with 5-year DSS being highest in married patients (88.4%) and lowest in widowed individuals (77.0%).

Significant differences in OS and DSS were observed among marital status groups (*p* < 0.001, log-rank test) (Figs. 1, 2)**.** Married patients had the highest 5-year OS (71.5%), followed by divorced (63.7%) and unmarried (61.9%) patients, whereas widowed patients had the lowest OS (40.9%) (Table 2)**.** At 10 years, this survival gap further widened, with OS rates of 50.6% in married, 44.5% in divorced, 43.9% in unmarried, and 16.8% in widowed patients. A similar trend was noted for DSS, with 5-year DSS being highest in married patients (88.4%) and lowest in widowed patients (77.0%).

### Univariate and multivariate cox regression analyses

The DSS Kaplan–Meier curve similarly revealed a survival disparity, as depicted in Fig. 2. Factors affecting DSS in univariate and multivariate Cox regression models are summarized in Table 3.

**Table 3:** Cox regression analysis according to disease specific survival

		Univariate analysis		Multivariate analysis	
		HR (95% CI)	*p*	HR (95% CI)	*p*
Marital status	Married	Reference	< 0.001	Reference	0.010
	Unmarried	1.99 (1.66–2.39)	< 0.001	1.29 (0.99–1.67)	0.058
	Divorced	1.75 (1.37–2.22)	< 0.001	1.61 (1.19–2.17)	0.002
	Widowed	2.04 (1.56–2.67)	< 0.001	1.30 (0.86–1.97)	0.209
Age	≤ 50	Reference	0.243		
	50–64	0.85 (0.65–1.12)	0.246		
	65 ≥	0.98 (0.76–1.26)	0.853		
Race	White	Reference	< 0.001	Reference	0.466
	Black	1.47 (1.21–1.78)	< 0.001	1.01 (0.77–1.31)	0.957
	Other	0.94 (0.67–1.33)	0.728	0.74 (0.45–1.20)	0.221
Place of Residence	Metro ≥ 1 M	Reference	0.496		
	Metro 250 K-1 M	0.95 (0.78–1.15)	0.608		
	Metro < 250 K	0.99 (0.74–1.35)	0.978		
	Non-Metro Adj	1.26 (0.95–1.66)	0.105		
	Non-Metro Non-Adj	1.07 (0.74–1.55)	0.714		
Median Houesehold Income	< $50,000	Reference	0.142		
	$50,000-$75,000	1.24 (0.99–1.56)	0.064		
	> $75,000	1.11 (0.94–1.30)	0.227		
Sequence	Only one primary cancer	Reference		Reference	
	2 or more primary cancer	0.66 (0.56–0.79)	< 0.001	0.89 (0.71–1.16)	0.318
Histology	IDC	Reference			
	Others	1.90 (1.58–2.28)	< 0.001	0.75 (0.47–1.20)	0.234
Grade	1	Reference	< 0.001	Reference	< 0.001
	2	0.30 (0.21–0.42)	< 0.001	1.20 (0.75–1.93)	0.443
	3	0.46 (0.38–0.54)	< 0.001	2.49 (1.55–3.98)	< 0.001
ER	Positive	Reference		Reference	
	Negative	3.05 (2.22–4.19)	< 0.001	2.01 (0.47–8.51)	0.344
PR	Positive	Reference			
	Negative	2.22 (1.79–2.75)	< 0.001	1.45 (1.04–2.02)	0.029
HER2	Negative	Reference			
	Positive	1.65 (1.33–2.05)	0.003		
Molecular subtype	Luminal A	Reference	< 0.001	Reference	0.674
	Luminal B	1.73 (1.39–2.17)	< 0.001	1.18 (0.90–1.54)	0.239
	HER2 rich	1.81 (0.81–4.05)	0.150	1.02 (0.17–6.0)	0.981
	TNBC	4.44 (3.06–6.43)	< 0.001	1.27 (0.26–6.20)	0.770
T stage	T1	Reference	< 0.001	Reference	< 0.001
	T2	3.75 (2.96–4.75)	< 0.001	2.43 (1.71–3.45)	< 0.001
	T3	7.40 (5.08–10.77)	< 0.001	2.28 (1.36–3.82)	0.002
	T4	10.63 (8.15–13.87)	< 0.001	1.95 (1.29–2.96)	0.002
N stage	N0	Reference	< 0.001	Reference	0.049
	N1	2.46 (2.04–2.97)	< 0.001	1.33 (1.03–1.73)	0.032
	N2	3.12 (2.40–4.05)	< 0.001	1.28 (0.87–1.89)	0.205
	N3	5.07 (3.87–6.64)	< 0.001	1.69 (1.13–2.52)	0.011
AJCC Stage	1	Reference	< 0.001	Reference	< 0.001
	2	3.24 (2.40–4.38)	< 0.001	1.26 (0.81–1.96)	0.312
	3	6.76 (4.95–9.22)	< 0.001	2.24 (1.33–3.78)	0.002
	4	40.0 (29.92–53.48	< 0.001	7.59 (4.52–12.75)	< 0.001
Surgery	BCS	Reference	< 0.001	Reference	< 0.001
	Mastectomy	1.20 (0.57–2.53)	0.641	0.66 (0.23–1.95)	0.453
	Not performed	11.15 (5.26–23.63)	< 0.001	1.79 (0.59–5.49)	0.307
Radiotherapy	Yes	Reference			
	No	0.97 (0.83–1.14)	0.726		
Chemotherapy	Yes	Reference		Reference	
	No	0.79 (0.68–0.92)	0.002	1.14 (0.91–1.43)	0.241
Systemic therapy	Yes	Reference			
	No	2.47 (2.12–2.86)	< 0.001	1.14 (0.87–1.49)	0.348

Univariate Cox regression analysis demonstrated that marital status was significantly associated with OS (p < 0.001). Compared to married patients, unmarried individuals had a 1.40 times higher risk of mortality (HR = 1.404, 95% CI: 1.243–1.586, *p* < 0.001), divorced patients had a 1.29 times higher risk (HR = 1.291, 95% CI: 1.099–1.517, *p* = 0.002), and widowed patients had the highest mortality risk with a 2.59 times higher risk (HR = 2.594, 95% CI: 2.246–2.996, *p* < 0.001).

A similar pattern was observed for DSS, where widowed patients had a 2.04 times higher risk of disease-specific mortality (HR = 2.037, 95% CI: 1.557–2.666, *p* < 0.001). Multivariate Cox regression analysis confirmed that marital status remained a significant independent predictor of survival after adjusting for clinical and demographic variables (*p* < 0.001). Unmarried patients had a 1.41 times higher risk of mortality (HR = 1.410, 95% CI: 1.193–1.667, *p* < 0.001), whereas divorced patients had a 1.30 times higher risk (HR = 1.305, 95% CI: 1.069–1.594, *p* = 0.009). Widowed patients exhibited the highest mortality risk, with a 1.72 times higher risk (HR = 1.724, 95% CI: 1.421–2.092, *p* < 0.001).

DSS analysis showed similar trends, with divorced patients having a 1.61 times higher risk (HR = 1.608, *p* = 0.002) and widowed individuals a 1.30 times higher risk (HR = 1.303, *p* = 0.010). Although unmarried patients showed a trend toward worse DSS, this finding did not reach statistical significance (HR = 1.285, *p* = 0.058).

## Discussion

Our findings indicate that marital status significantly influences survival outcomes in MBC, with widowed patients experiencing the worst prognosis among all groups (Tables 2 and 3)**.** This observation highlights the important role of social and psychological factors in cancer outcomes, particularly for widowed individuals who may face unique challenges that adversely affect their prognosis. Kaplan–Meier survival analysis results (Figs. 1 and 2**)** confirm that widowed patients exhibit significantly lower overall and disease-specific survival rates. Economic stability and healthcare access disparities among marital status groups may contribute to these findings (Table 1)**.**

Our study also found that the histologic grade of breast cancer was lower in married men compared to unmarried men. Since tumor grade is a well-established prognostic factor, this finding suggests a potential link between survival and disease biology. Given that lower-grade tumors are generally associated with better survival outcomes, the higher prevalence of low-grade tumors in married men may be one factor contributing to their improved survival.

The observed differences in tumor characteristics across marital status groups may further reflect disparities in healthcare access, health literacy, and health-seeking behavior. For example, unmarried or widowed individuals may be less likely to undergo regular medical evaluations or respond promptly to symptoms, potentially leading to delayed diagnosis and more advanced disease. While these findings are descriptive and do not establish causality, they underscore the role of psychosocial and socioeconomic factors in influencing disease presentation. Additionally, unmeasured variables such as comorbidities, healthcare accessibility, or underlying health behavior patterns—none of which are captured in the SEER database—may also contribute to these differences. Future studies integrating biological, psychosocial, and systemic variables are warranted to further elucidate these relationships.

Although the majority of patients across all marital status groups were diagnosed at an early stage (Stage I–II), we observed substantial variation in disease-specific survival (DSS), particularly among widowed patients. This apparent paradox may be explained by a combination of clinical, biological, and psychosocial factors. Widowed patients were markedly older (mean age: 78.7 years), which likely reflects greater physiological vulnerability and a higher burden of age-related comorbidities. While DSS aims to isolate cancer-specific mortality, registry-based datasets such as SEER have the potential for misclassification and residual confounding. In addition, widowed patients were significantly less likely to receive standard treatment; for instance, more than 25% did not undergo surgery, and only 18.8% received chemotherapy. Such under-treatment may lead to poorer outcomes even in early-stage disease. From a tumor biology perspective, widowed patients exhibited higher rates of high-grade tumors, hormone receptor negativity, and aggressive molecular subtypes, suggesting more biologically unfavorable disease characteristics despite similar clinical staging. Lastly, psychosocial stress and lack of support in widowed individuals may contribute to worse outcomes by affecting treatment adherence and cancer progression through neuroendocrine–immune mechanisms.

In our study, we found that unmarried, widowed, or divorced men were more likely to be diagnosed at an advanced stage compared to their married counterparts. Several factors may contribute to this observation. Firstly, spouses—particularly women—may be more likely to notice changes in their husband's breast, prompting earlier medical consultation. Secondly, men may underestimate or neglect breast-related symptoms, leading to delays in seeking medical attention. Additionally, married individuals may receive encouragement and support from their spouses to seek medical care earlier. These findings raise the hypothesis that marital status may influence the timeliness of diagnosis in male breast cancer; however, this could not be directly evaluated in our study due to the absence of data on symptom-to-diagnosis intervals. As such, this interpretation should be viewed as speculative and warrants further investigation.

### Social ısolation and lack of support

Unmarried patients, including those who are widowed, have been found to have a significantly higher risk of metastatic cancer, inadequate treatment, and cancer-related mortality compared to married patients. The poorer prognosis in widowed patients compared to unmarried patients may not be solely attributable to social isolation but could also result from advanced age and a higher burden of comorbidities, which are more prevalent in this group. While social isolation remains an important determinant, the strong association between advanced age and mortality risk in widowed patients suggests that physiological decline, multimorbidity, and frailty may be key contributing factors. Although widowed patients had the highest mortality risk, multivariate analysis showed a non-significant association (*p* = 0.209)**,** indicating that additional unmeasured factors such as comorbidities and healthcare access might influence survival. In addition to social isolation, advanced age appears to be a key determinant of poorer survival outcomes in widowed patients, given its association with increased comorbidities and reduced physiological resilience [11]**.** Widowed patients are often at higher risk of social isolation compared to other marital status groups. The absence of a partner or close support system can result in reduced adherence to treatment and limited access to healthcare services. Social isolation has been associated with higher rates of depression and anxiety, both of which are known to negatively impact cancer survival [12, 13]**.** Socially isolated individuals with breast cancer have an increased risk of all-cause mortality, and the literature also indicates a higher cancer-specific mortality rate in breast cancer patients due to social isolation [13]**.** In widowed patients, the lack of emotional and logistical support may hinder their ability to manage complex treatment regimens and maintain consistent follow-up care.

Studies have shown that strong social support networks are associated with improved cancer outcomes, as they facilitate better coping mechanisms and adherence to treatment protocols [12, 14]**.** In contrast, widowed individuals are more vulnerable to the psychological burden of cancer, leading to increased stress and reduced resilience, which may further compromise their prognosis [11–14]**.**

### Economic challenges and healthcare access

In unmarried or widowed men, the presence of more advanced disease compared to married men is likely attributable to reduced access to healthcare services. Economic stability plays a crucial role in cancer care, and widowed patients often face greater financial difficulties than their married counterparts. Reduced household income and limited access to health insurance can lead to delayed diagnosis and suboptimal treatment [15]**.** These factors may contribute to the higher proportion of advanced-stage disease observed in widowed patients in our study.

The financial burden of cancer treatment, coupled with the absence of a supporting partner, may deter widowed patients from seeking timely and adequate care. Addressing these economic and healthcare access disparities is essential to improving survival outcomes in this high-risk population [2, 15]**.**

Socioeconomic disadvantages—such as lower income and residence in non-metropolitan areas—were more prevalent among widowed patients and may partially account for their poorer prognosis. These factors can significantly impact access to healthcare services, delay diagnosis, and hinder adherence to treatment. This reinforces the multifactorial nature of marital status as a prognostic determinant in MBC.

### Psychoneuroimmunological effects

Observational studies conducted in individuals with various cancers at both early and advanced stages suggest that chronic stress and psychological distress are associated with impaired immunoprotective responses. Factors such as social support, which is thought to act as a buffer against the effects of chronic stress, are associated with stronger immunoprotective responses [5, 13, 14, 16]. The physiological mechanisms underlying the survival disparity in widowed patients may involve stress-induced immunosuppression and altered neuroendocrine responses. Human and animal studies have shown that chronic stress suppresses various aspects of protective immunity, including cell-mediated immunity, antibody responses, immune cell proliferation, graft rejection, T-cell and NK cell–mediated antiviral responses, and macrophage-mediated antimycobacterial activity. NK cell activity, cell-mediated immunity, and other types of immune responses are crucial for tumor surveillance and for preventing tumor progression, spread, and metastasis [16]. Chronic stress associated with bereavement and social isolation can suppress natural killer (NK) cell activity, which plays a vital role in identifying and destroying cancer cells. Additionally, prolonged stress can disrupt hormonal balance, particularly through elevated cortisol levels, further weakening the immune system’s ability to combat cancer progression [16].

Research has also highlighted the potential role of oxytocin, a hormone released during social bonding, in reducing stress responses and promoting immune function. The absence of social interactions in widowed individuals may limit the protective effects of oxytocin, leaving them more susceptible to disease progression [15–17].

### Clinical ımplications and recommendations

Our findings emphasize the important role of marital status as an independent prognostic factor in MBC, with widowed patients demonstrating the poorest survival outcomes (Tables 2 and 3)**.** These patients face nearly double the risk of mortality compared to their married counterparts, highlighting the urgent need for targeted interventions. Integrating psychosocial support programs, mental health screening, and patient navigation services into standard oncology care could help bridge this survival gap. Early identification of widowed patients as a high-risk population and the provision of tailored support may significantly improve their prognosis.

Collaboration among oncology, psycho-oncology, and social work services is essential for implementing structured support mechanisms such as peer mentorship programs, targeted mental health interventions, and community-driven assistance initiatives. Developing structured social support programs and improving healthcare access, particularly for widowed and unmarried patients, could mitigate the negative impact of social isolation and improve survival outcomes. Future research should focus on evaluating the effectiveness of these interventions in prospective trials. Expanding psychosocial resources and incorporating mental health services into routine oncology care will be key steps toward reducing survival disparities and improving quality of life for vulnerable MBC patients.

The relative rarity of MBC limits the feasibility of a prospective study with sufficient sample size and power to detect any impact of marital status on stage and survival. In this study, the use of the SEER database helped overcome the sample size limitation. Although our study identifies marital status as a prognostic factor in male breast cancer, the absence of direct psychological and social support data in SEER limits our ability to determine causality. Moreover, marital status was recorded only at the time of diagnosis and does not reflect any changes that may have occurred during follow-up, such as divorce, remarriage, or widowhood. This may result in misclassification bias and could dilute or obscure the true effect of marital status on survival outcomes. The SEER database does not include information about household composition, caregiving dynamics, or familial support structures, all of which may influence patient behavior, treatment adherence, and survival outcomes. The absence of these variables may confound the observed associations between marital status and prognosis. Furthermore, the SEER database lacks detailed information on chemotherapy regimens, hormonal therapy adherence, and duration or compliance with systemic treatment. This limitation restricts the ability to control for treatment-related confounders that may influence survival outcomes. Additionally, while tumor characteristics varied significantly across marital status groups, these associations may be partly influenced by unmeasured confounders, including healthcare access, health literacy, or comorbidities, which are not fully captured in the SEER database. A potential limitation is the very small number of widowed patients aged < 50 years (*n* = 2). Although age was not a statistically significant factor in the DSS model, this subgroup's small size may have contributed to reduced precision in the hazard ratio estimates for widowed patients, as evidenced by the wider confidence interval. Future studies with larger subgroup samples may provide more definitive insights. Another limitation of the study is the use of different AJCC staging editions over the study period, as per SEER database conventions. The 6th edition was applied between 2010 and 2015, the 7th edition between 2016 and 2017, and the 8th edition from 2018 onward. As the definitions of stage categories have evolved—particularly with the incorporation of biological markers in the 8th edition—this may introduce misclassification bias in staging-related analyses. Consequently, comparisons across the full cohort based on AJCC stage should be interpreted with caution.

In conclusion, widowed patients represent a particularly vulnerable group with significantly poorer survival outcomes. Addressing their unique psychosocial and economic challenges should be a priority in clinical practice to improve overall survival and quality of life for this population.

## Conclusion

These findings underscore the significant impact of marital status on survival outcomes in MBC patients. Married individuals exhibit the most favorable survival outcomes, while widowed patients face a notably higher risk of mortality. The nearly doubled mortality risk in widowed patients compared to their married counterparts highlights the urgent need for targeted psychosocial and clinical interventions. Integrating mental health resources, psychosocial support programs, and patient navigation services into routine oncology care could significantly improve outcomes for high-risk populations.

Future research should prioritize evaluating the effectiveness of structured support mechanisms such as peer mentorship programs, targeted mental health interventions, and community-driven assistance initiatives in prospective trials. Expanding access to comprehensive cancer care and enhancing social support systems are essential steps toward reducing survival disparities. Addressing these challenges is not only vital for improving survival outcomes but also represents a critical step toward achieving equitable cancer care for all.

## Data Availability

The data that support the findings of this study are available from the corresponding author upon reasonable request.
